# Supplementary material for: Vancomycin-Resistant Enterococci (VRE) in Nigeria: The First Systematic Review and Meta-Analysis
Source: Antibiotics (Basel). 2020 Sep 1;9(9):565. doi: 10.3390/antibiotics9090565 (PMC7558171; doi:10.3390/antibiotics9090565)
Supplement: Supplementary file 1 [file antibiotics-09-00565-s001.zip › Supplemetary file 2.pdf]

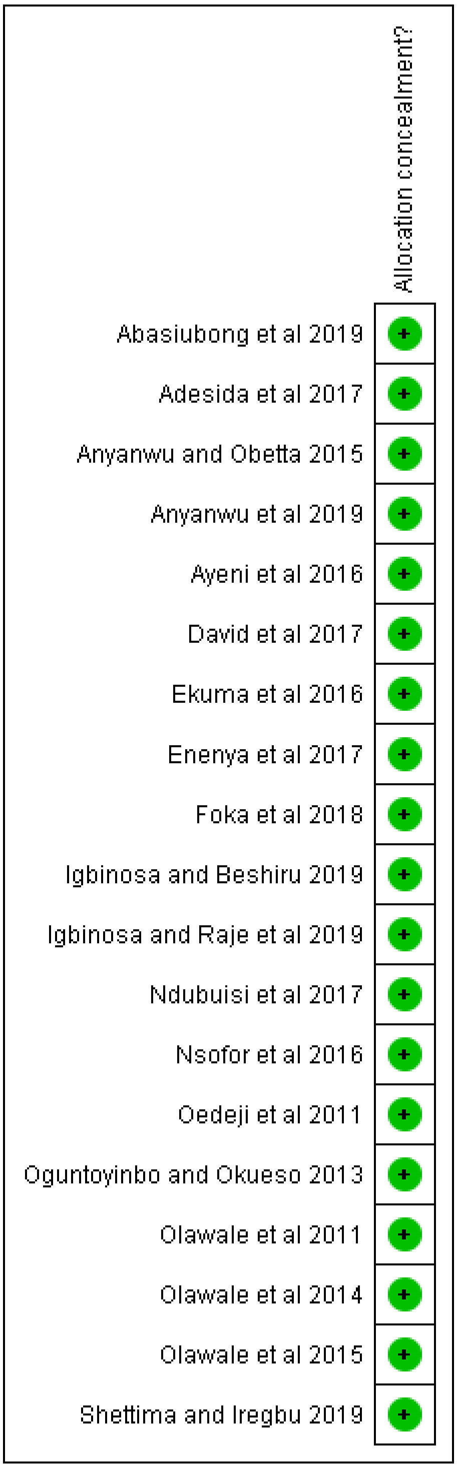

Figure S1: Risk of bias summary of VRE studies in Nigeria

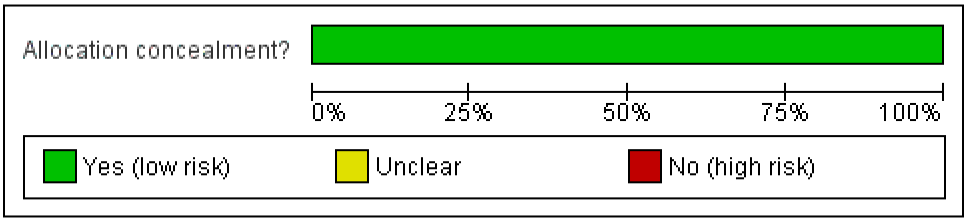

Figure S2: Risk of bias graph of VRE studies in Nigeria
